# Detailed Thermal Characterization of Acrylonitrile Butadiene Styrene and Polylactic Acid Based Carbon Composites Used in Additive Manufacturing

**DOI:** 10.3390/polym12122960

**Published:** 2020-12-11

**Authors:** Zoltan Ujfalusi, Attila Pentek, Roland Told, Adam Schiffer, Miklos Nyitrai, Peter Maroti

**Affiliations:** 1 Department of Biophysics, Medical School, University of Pécs, Szigeti str. 12, H-7624 Pécs, Hungary; zoltan.ujfalusi@aok.pte.hu (Z.U.); miklos.nyitrai@aok.pte.hu (M.N.); 2 3D Printing and Visualization Centre, University of Pécs, Boszorkany str. 2, H-7624 Pécs, Hungary; pentekattilaistvan@gmail.com (A.P.); told.roland@pte.hu (R.T.); 3 Department of Technical Informatics, Faculty of Engineering and Information Technology, University of Pécs, Boszorkany str. 2, H-7624 Pécs, Hungary; schiffer.adam@mik.pte.hu; 4 MTA-PTE Nuclear-Mitochondrial Interactions Research Group, H-7624 Pécs, Hungary; 5 Medical Simulation Education Center, Medical School, University of Pécs, Szigeti str. 12, H-7624 Pécs, Hungary

**Keywords:** PLA, ABS, carbon, resistance, DSC, thermogravimetry, composite, additive manufacturing, biomedical sensors

## Abstract

Currently, 3D printing is an affordable technology for industry, healthcare, and individuals. Understanding the mechanical properties and thermoplastic behaviour of the composites is critical for the users. Our results give guidance for certain target groups including professionals in the field of additive manufacturing for biomedical components with in-depth characterisation of the examined commercially available ABS and PLA carbon-based composites. The study aimed to characterize these materials in terms of thermal behaviour and structure. The result of the heating-cooling loops is the thermal hysteresis effect of Ohmic resistance with its accommodation property in the temperature range of 20–84 °C for ESD-ABS and 20–72 °C for ESD-PLA. DSC-TGA measurements showed that the carbon content of the examined ESD samples is ~10–20% (*m*/*m*) and there is no significant difference in the thermodynamic behaviour of the basic ABS/PLA samples and their ESD compounds within the temperature range typically used for 3D printing. The results support the detailed design process of 3D-printed electrical components and prove that ABS and PLA carbon composites are suitable for prototyping and the production of biomedical sensors.

## 1. Introduction

3D printing is reshaping the world of prototyping and production. It is present in architecture, arts, industry, healthcare, and even everyday households [1,2]. As a disruptive technology, additive manufacturing can decrease the cost, production time, and the rate of environmental burden as well. The most commonly used desktop FFF (Fused-Filament-Fabrication) 3D printers provide an unmatched solution in conceptualization, modelling, rapid prototyping even in small-series production [3,4,5,6,7]. The range of usable materials are relatively wide. Almost any extrudable thermoplastic polymer or their composite can be printed out with desktop devices [8,9]. Using machines with two or more extruders allow the users to create complex objects, containing multiple materials. The feature can significantly reduce production time and costs, which are key factors in research work or a product development process. Previous studies have investigated complex models fabricated with additive manufacturing, where interlayer adhesion is a key factor, determining the characteristics of the printed object. It has been revealed that the printing parameters like nozzle temperature, bed temperature, and layer height also has a significant effect on fracture behaviour [10] as well as the combination of the different materials [11,12]. In addition, the treatment of the printed objects can modify layer adhesion. As a post-processing method, heat or chemical treatment can be applied in order to modify the surface or structure characteristics [12], and these observations are essential in case of functional prototyping. Therefore, the detailed thermal and structural characterization of the materials are essential to better understand their potential application.

PLA (polylactic acid) and ABS (acrylonitrile butadiene styrene) are among the most widely used 3D printing materials [4]. PLA is a biocompatible and biodegradable thermoplastic polyester and can be obtained by the condensation of lactic acid or by ring-opening polymerization of lactide. ABS is a common thermoplastic polymer with an amorphous structure, produced by polymerizing styrene and acrylonitrile, in the constant presence of polybutadiene. Both materials are used in their neat form, but numerous ABS-based and PLA-based composites are available. PLA can be blended with cork or wood-flour, which can be used as a lightweight and biodegradable material in rapid prototyping, but it can significantly decrease the mechanical properties [13,14,15,16]. The mechanical behaviour of PLA-graphene and PLA-carbon composites are also investigated previously [14,15], as well as fiber or metal reinforcement techniques [14]. ABS can also be used as a base material for different composites containing stainless steel [17], glass-fibers [18], or graphene [15]. Adding these plasticizers and compatibilizers to ABS and PLA base materials results in a significant change in material characteristics and functionality. Therefore, the rigorous and detailed evaluation is necessary.

Most of the prototypes and models require electric components, mainly in the field of industry and biomedical applications [19]. Previous studies have shown that it is possible to create built-in sensors, antennas, or even electrically conductive parts using FFF 3D printing technology with the aid of different ABS-based or PLA-based carbon composites [3,19,20,21,22]. Despite the extensive research of the biomedical applications of the mentioned materials, the thermal and hysteretic behavior of ESD (electrostatic discharge)-ABS and ESD-PLA have not been examined before. For certain applications, it is a great advantage if a new composite has better properties than its predecessors, e.g., the increased thermal and mechanical stabilities [23]. In other cases, lower melting temperature and decreased thermal stability can be a huge advantage like the application of polycaprolactone (PCL) as a flexible scaffold in tissue engineering [24]. With DSC-TGA (differential scanning calorimetry with thermogravimetry analysis) examinations, we can map the thermal properties of different composite materials very efficiently and accurately. The extent to which different additives change certain properties of the original composite (melting point, crystallization, and recrystallization temperatures, etc.) can also be determined. Since DSC examinations of individual composite materials is arguably one of the most important test methods, such measurements have been performed on both ESD composites to get a detailed picture about the temperature profiles and to determine the carbon content of the samples. It is also well known that the resistance of electrically conductive composite materials (ESD-ABS and ESD-PLA) strongly depends on the temperature. In our measurements, the heating-cooling periodic temperature excitation loops resulted in lagged resistance change as an output, so there is a hysteresis relation between the temperature and the electrical resistance (ER) *R*-*T* curves. Here, we provide a thorough and detailed analysis of ESD composites, revealing the mechanical properties, the thermal hysteretic behaviour, and the detailed temperature dependence. Our unique experimental setup for resistance measurements and the high sensitivity DSC-TG measurements provide valuable data for industrial, healthcare, and private users. This is the first time such a powerful combination of tools is applied to get a more accurate picture about these compounds. In addition, the paper describes a potential and promising biomedical-related use case. With the detailed thermal and electrical characterisation of the materials, the different functional models and prototypes can be designed with more accuracy and precision, which is essential in biomedical engineering. 3D printing has already proven its indispensable place in the fields of medical and biomedical applications [25]. Thus, the proper characterisation of the ESD compounds can be beneficial to these fields.

## 2. Materials and Methods

### 2.1. Composite Materials Used

#### ESD-PLA Samples

We used ESD-PLA filament samples made by Filamania Ltd. (Jozsef Attila Street 57, H-3527 Miskolc, Hungary) and the transparent, regular PLA we used for the control experiments was manufactured by Filamania Ltd. (Jozsef Attila Street 57, H-3527 Miskolc, Hungary). The average filament diameter was 1.75 ± 0.05 mm. According to our recent study, test bars printed with X printing orientation and 100% infill density had an impact strength of 12.32 ± 0.57 kJ/m^2^ and the flexural strength was 48.45 ± 0.98 MPa. The value of tensile strength is measured to be 25.88 ± 0.88 MPa. The Shore D values were 67.54 ± 1.25. None of the samples had any kind of pre-treatments. They were used straight out of the packaging.

### 2.2. ESD-ABS Samples

ESD-ABS filament samples were applied for all experiments made by HobbyKing^®^ (Lucky Stuff Limited, 18 Elmsett Airfield, Elmsett, IP7 6LN, Ipswich Suffolk, UK). We used dark blue, pigmented, regular ABS for the control experiments made by HobbyKing^®^. The material printed in the X orientation with 100% infill density, had a 15.37 ± 1.66 kJ/m^2^ impact strength value, while the flexural strength was 39.18 ± 1.72 MPa with the same printing parameters. The tensile strength was 19.15 ± 0.30 MPa. Shore D measurements of the values were 64.2 ± 1.00. None of the samples had any kind of pre-treatment. They were used straight out of the packaging.

### 2.3. Resistance Measurements

**Figure 1:**
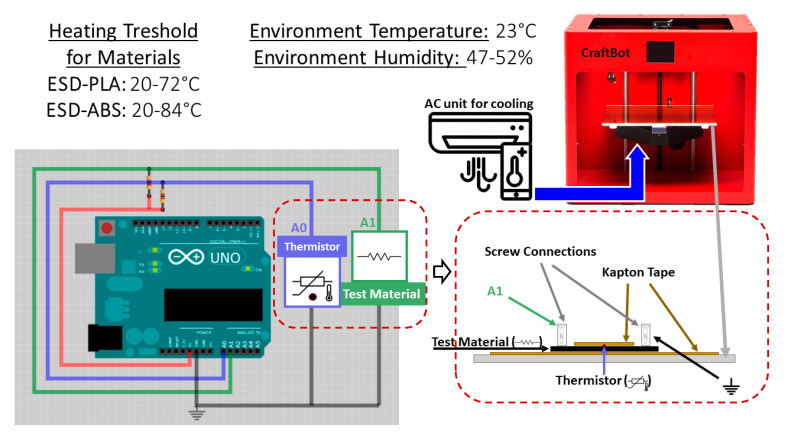
The resistance and temperature measurement setup with the data acquisition unit. The test specimen was insulated and fixed to the printing bed with Kapton tape. On the upper side, between the tape and test specimen, a thermometer was inserted, and the test specimen is connected to the voltage divider on both sides.

The ESD-PLA was printed with a hot end of 210 °C on a 50 °C preheated tray, while ESD-ABS was printed with a hot end of 240 °C on a 110 °C tray. All specimens and ESD-ABS temperature sensor-based prosthetic fingertips are printed in direction “X” (Figure S5). Both sample types were printed with a 0.4 mm nozzle diameter at 60 mm/second printing speed using a Craftbot Plus desktop 3D printer (Craftunique Ltd., Ilka Street 50, 1143 Budapest, Hungary). All the printed test specimens were conditioned at ambient temperature for at least 4 h before testing, according to the ISO 291 standard. Between each measurement, the test specimens were conditioned at ambient temperature for at least 24 h. (5 × 50 × 0.8) mm specimens were printed from these electrically conductive materials with parallel infill and a 200 μm layer thickness. From each of the materials, five specimens were printed with a 0.8 mm height (four layers). The variation of temperature was obtained by a thermostat. The sketch of the apparatus with the thermostat and the data acquisition system is shown in Figure 1.

The temperature of the specimen is measured with a high temperature Teflon thermometer with a resistance of 100 Ω (NTC 3950), which has a standard uncertainty of 0.1 °C. The data acquisition was performed, and the data were collected by an Arduino Uno with 1000 bits ADC. The sampling frequency was $f_{s}=10\;\mathrm{Hz}$.

In the experiments, the ESD-ABS samples were heated from 20 °C to 84 °C and then cooled back to 20 °C, thus completing an entire cycle. The temperature was increased monotonically and decreased according to the natural cooling at an ambient temperature. Five temperature increase-decrease cycles were measured in a series. The accommodation can be characterized by exponentially decreasing resistance curves with asymptotes that can be calculated from the heating loops at the same temperature value. The defined measurement has been repeated with different specimens (five ESD-ABS and five ESD-PLA samples) and all measurements have been repeated after 24 h. Repeatability has high importance because it is a good indicator of any changes in the structure of the composite materials after the heating-cooling cycles.

To present the application of the conductive ESD-ABS and ESD-PLA, we have implemented a thermometer made of composites in the index fingertip of the model arm. The sensors and the prosthesis fingers were printed with the print settings of the specimens used during the measurements. The thermometer sensor was designed with two layers (400 µm) below the surface of the finger so that environmental disturbances would not affect the operation. Since a voltage divider is connected to the two endpoints of the sensor, an Arduino Uno was used for the resistance measurement. At a pre-defined resistance value, the connected indicator LED (light emitting diode) is lit.

### 2.4. DSC-TGA Measurements

These measurements were performed with a LabSys Evo instrument (Setaram Ltd., 7 Rue de l’Oratoire, 69300 Caluire-et-Cuire, France) to analyse certain properties of different composite materials. The scale accuracy, temperature, and enthalpy calibration of the equipment was recently performed officially by Setaram. DSC and thermogravimetry analysis (DSC-TGA) measurements were carried out under 100 mL/min nitrogen atmosphere and the mass of each sample was set in the range from 4.5 mg to 8.0 mg. For measurements up to 300 °C, V = 75 µL, uncovered aluminium crucibles (Setaram Ltd., 7 Rue de l’Oratoire, 69300 Caluire-et-Cuire, France) were used while, for experiments up to 600 °C, we used V = 100 µL and uncovered Al_2_O_3_ crucibles (made by Setaram Ltd., 7 Rue de l’Oratoire, 69300 Caluire-et-Cuire, France). The rate of the sample cooldown was appropriate for the measurements. The application of any external cooler was not necessary. DSC-TGA measurements were performed at two distinct temperature ranges where the temperature was increased at a rate of 10 °C/min in both cases. Thermograms with mass change were recorded in the range of (30–300–30) °C, which is a bit above the peak temperature generally used in 3D printing (~215 °C typically for PLA and ~250 °C for ABS samples) or further above to 600 °C (starting from 30 °C) for certain measurements. These later measurements proved to be the best to record the maximal achievable mass decrease while keeping the extra carbon content safe from deterioration [26].

### 2.5. Carbon Content of ESD Samples

Though the suppliers know the chemical compositions of their products, in many cases, that information is not shared with the users. We applied two distinct methods here to determine the content of carbon filler in the examined composites. In Method 1, there were two separate thermogravimetric analyses (TGA) performed with the same type of host material, non-ESD and ESD, respectively. The raw PLA and ABS samples were pyrolyzed under N_2_ atmosphere in the temperature range of 30 °C to 600 °C and then the same process was repeated with the ESD samples. The mass of the residuals in the crucibles at the end of the analyses were measured and then the mass of the residue of the raw host material was subtracted from the residue mass of the ESD sample to get the carbon content of the given compound. The result we got this way was expressed as the percentage of the initial sample mass (m/m%) similarly to literature data [27]. Because of the applied high temperature, the application of Al_2_O_3_ crucibles was necessary. In Method 2, we applied several solvents to dissolve and remove all the plastic components wrapping around the carbon particles and then weigh the indissoluble carbon that remained. This method was not applicable for ESD-ABS samples as we were not able to completely dissolve ABS in any of the used compounds. The applied organic solvents were acetone, butanone (methyl ethyl ketone (MEK)), and tetrahydrofuran (THF). All these organic compounds rapidly dismantled the ABS composites but were not able to fully dissolve the plastic components even after one month of soaking at room temperature (~22 °C). For ESD-PLA samples, 6N KOH proved to be an excellent solvent that dissolved PLA quickly around the carbon particles (12 h were enough to completely dissolve the host material). The KOH was then carefully removed, and the mass of the remaining carbon-powder was determined.

### 2.6. Data Analysis and Figures

Data evaluation were performed using the OriginLab Origin^®^ 2021 (version 9.8.0.200) (OriginLab Corporation, One Roundhouse Plaza, Suite 303, Northampton, MA, USA) and Microsoft Excel (version 2002) (Microsoft Corporation, One Microsoft Way, Redmond, WA, USA) software. The figures were made using OriginLab Origin^®^ 2021 and Microsoft PowerPoint (version 2002) (Microsoft Corporation, One Microsoft Way, Redmond, WA 98052-7329, USA) software.

## 3. Results

### 3.1. Resistance of Composites

**Figure 2:**
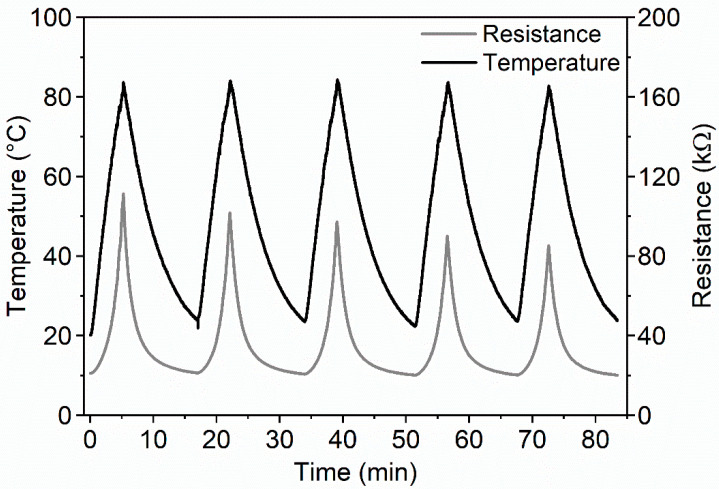
Exciting temperature curves and the measured lagged resistance curves of ESD-ABS specimen No 1. The asymmetry of increasing and decreasing resistance curves causes the hysteresis effect and the decreasing turning points of the ER cycles causing the accommodation property.

**Figure 3:**
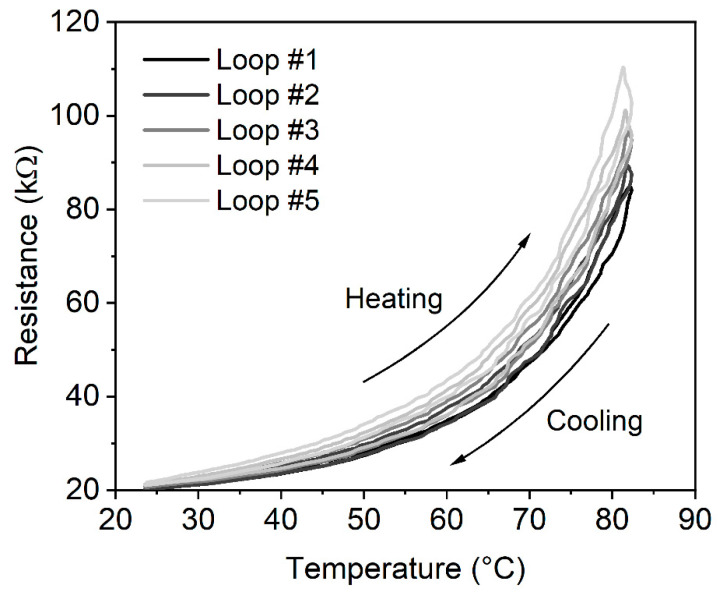
The hysteretic behaviour of the five *R*-*T* curves of the ESD-ABS composite material. The figure clearly shows the alteration between the heating and cooling loops. The accommodation property can also be examined from the turning points.

During resistance tests, five ESD-ABS and five ESD-PLA specimens were examined (marked as No.1 … No.5, respectively). The measured temperature and resistance curves of ESD-ABS composite material, specimen No. 1 are shown in Figure 2. The heating-cooling temperature curves are in the range of 20–84 °C with the similar peak (turning point), the peaks are in °C: 83.68, 83.99, 84.32, 83.05, and 82.43, respectively. The measured resistance turning points are decreasing in kΩ: 111.3, 101.8, 95.95, 90.08, and 85.28, respectively. The heating-cooling asymmetry of the resistance curves is more significant than the asymmetry of the temperature curves. It causes the hysteresis property that can be seen in Figure 3. The first turning point is at ambient temperature, 20 °C, where all loops are identical. Reaching the turning point at a maximum temperature, the cooling curves are different. The temperature dependence *R*(*T*) is relatively limited around R=20 kΩ but significant at R=90 kΩ ($T_{heating}=78.24$ °C, $T_{cooling}=80.16{}^{\circ}C$) at the first loop.

### 3.2. Amount of Carbon in ESD Samples

**Figure 4:**
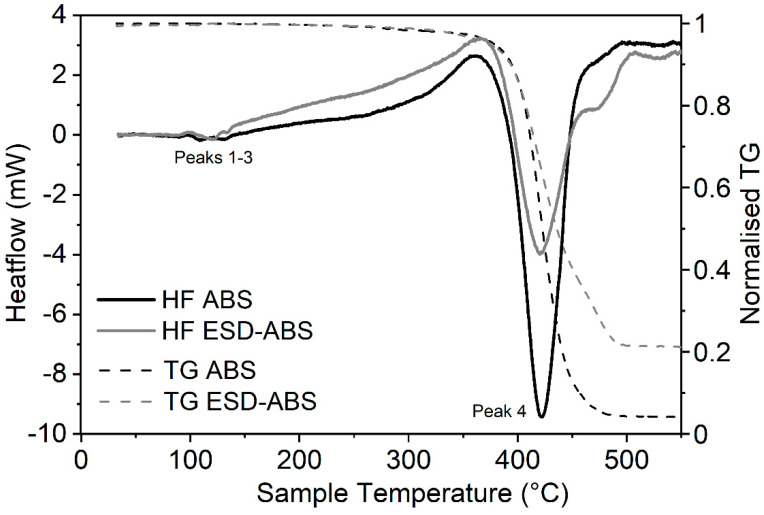
DSC heating curves of ABS and ESD-ABS (30–550 °C, solid lines) combined with TGA of the samples (30–550 °C, dashed lines) at a heating rate of 10 °C/min. On the heating curves, the exothermic process moves upwards. (HF—Heat flow. TG—decrease in mass during thermogravimetric analysis).

**Figure 5:**
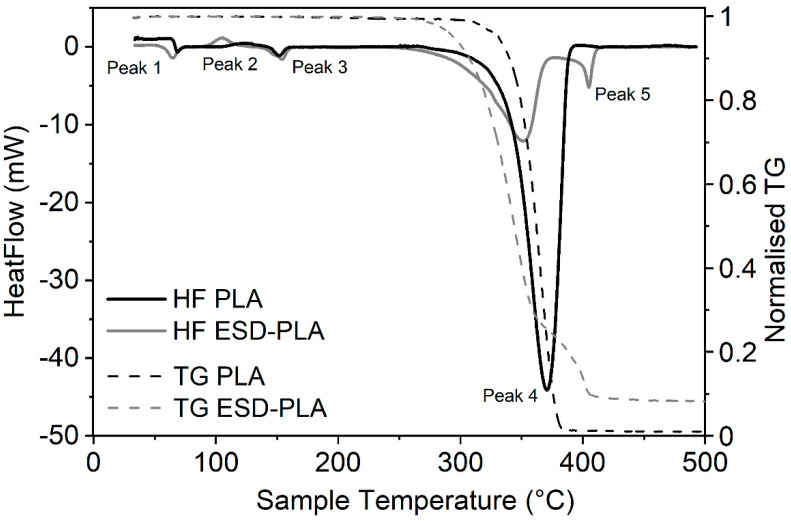
DSC heating curves of PLA and ESD-PLA (30–500 °C, solid lines) combined with TGA of the samples (dashed lines) at a heating rate of 10 °C/min. On the heating curves, the exothermic process moves upwards. (HF—Heat flow. TG—decrease in mass during thermogravimetric analysis).

ESD samples contain carbon to prevent the occurrence of static electricity in the printed models or we can use them as conductive printouts and test their resistance, as we have described above. The carbon filler content of certain ESD filaments can be an important data that is usually not mentioned in the datasheet of these products by the manufacturers. We applied two distinct methods, thermogravimetric analysis of the pyrolyzation of the composites and dissolution of the host component to determine the carbon content of the provided filament samples. All TGA measurements were combined with DSC signals to see how the carbon content affects the thermal properties of these filaments. The carbon content of ESD-ABS samples determined by TGA (Method 1 in Materials and Methods) was around 18.22% ± 1.69% (*m*/*m*) (Figure 4). In the case of ESD-PLA samples, the carbon content was determined to be 8.79% ± 3.33% (*m*/*m*) by TGA (Figure 5). The major decrease in mass nicely correlates with the decomposition peaks (Peak 4) of the corresponding DSC curves of both sample types (Figure 4 and Figure 5).

For ESD-PLA samples, the dissolution of PLA (Method 2 in Materials and Methods) could also lead to a good estimation of its carbon content, which is determined to be 10.14% ± 0.73% (*m*/*m*) this way. Though this is not the most elegant way to determine the dry mass of carbon filler added to the host component, the chemical treatment of composite materials is a well-known and standard practice for changing certain physical/mechanical properties, making the source material applicable for special use [12,28,29,30,31,32]. If we compare the carbon filler mass of ESD-PLA samples determined by the two different methods, the results are relatively close to each other, which suggests an estimated carbon content of 8%–10%. These results nicely correlate with literature data about the significantly better conductivity of ESD-ABS samples over the ESD-PLA ones [33].

### 3.3. Thermal Characterisation of Composites

The characterisation of the thermal properties of ESD-PLA and ESD-ABS samples were performed with DSC measurements. For both sample types, the glass transition (*T_g_*) phase is slightly more expressed in the case of carbon-free samples (Figure 4 and Figure 5).

Significant changes in temperature parameters is observable for the PLA samples at the onset of the glass transition while, for the ABS samples, there is a larger shift in the temperature parameters at the end of the same transition (Peak 1 in Table 1). The same tendency can be observed with the crystallization peaks (Peak 2 with *T_c_*), as the carbon-containing PLA sample reaches this phase at a significantly lower temperature (by ~21 °C) while, in the case of the ABS samples, the crystallization peak for the ESD sample is much closer to the starting point of the melting phase than that of the non-ESD one.

Interestingly, there is no significant difference in melting temperature data (*T_m_*) for any PLA or ABS sample. As we go above printing temperature, by far, the control PLA samples seem to be slightly more stable and have the temperature of decomposition (*T_d_*) at ~19 °C higher than ESD-PLA (Table 1). The calculated enthalpy change values (Δ*H*) for the decomposition phase (Peak 4) underline the decreased overall stability of the ESD-PLA samples. However, in the temperature range that is routinely used in 3D printing, the opposite tendency can be observed as slightly more energy required to achieve a certain phase for the ESD-PLA than necessary for the carbon-free PLA (Figure 5, Table 2, Figure S6).

The huge change in the enthalpy of Peak 4 of PLA samples cannot be fully explained with the roughly 10% carbon content even if we take the extra fifth peak into consideration in the case of ESD-PLA (Figure 5 and Table 2). The same tendency for Δ*H* is observable in the case of the ABS and ESD-ABS filaments as well for the decomposition phase (Peak 4). The crystallization phase is more expressed for the ESD-PLA than that of the PLA composite and it is matching well with literature data [36] while there is not that much difference observable in the case of the ABS samples. The recrystallisation phase (Peak 6) is much less prolonged for the ESD-PLA during sample cooldown, but that does not cause any significant change in the peak enthalpies (recorded only for samples heated up to a maximum of 300 °C) (Table 3).

Both control ABS and ESD-ABS samples have very similar values in the cooling phase apart from an extra peak observed for the ESD-ABS samples (Table 3).

## 4. Discussion

**Figure 6:**
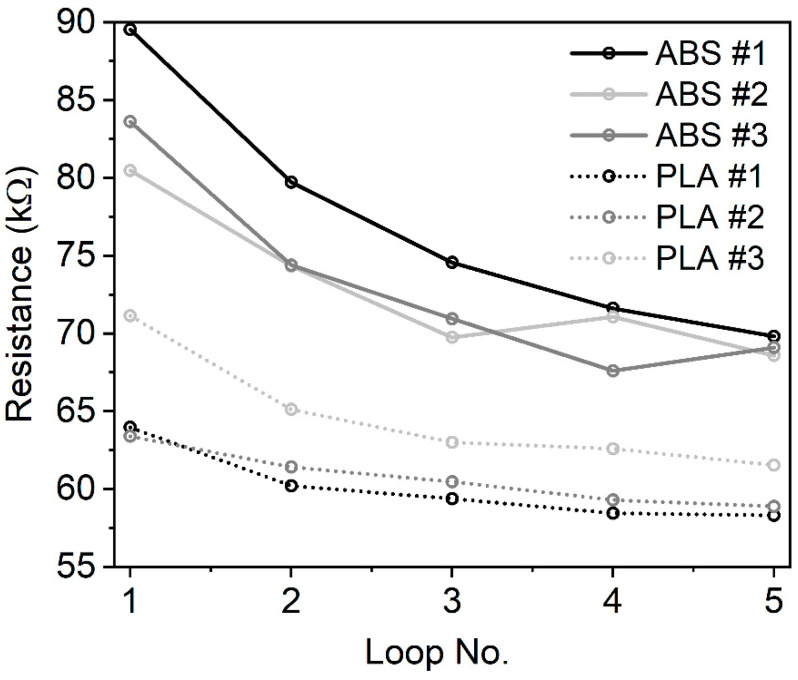
The resistance values in kΩ of the heating curve at the same temperature point $T_{p}$ (ESD-ABS $T_{p}=$ 78 °C, ESD-PLA $T_{p}=$ 68 °C) in different loops. It can be recognized that the *R* values are decreased exponentially at the fixed *T* values as the number of the excitation loops is increased.

Previous studies have proved that ABS-based and PLA-based carbon composites as thermoplastic polymers are suitable for manufacturing complex models containing the electrical part and components. The mechanical and structural characteristics are well known. In addition, information on electrical conductivity have been revealed recently, as well as structural characteristics determined by scanning electron microscopy [23,33]. Despite the intense research work in the field, the detailed thermal characterisation of the materials has not been carried out before. The results of the study give opportunity to professionals, crafters, and biomedical engineers to design conductive, built-in objects with defined functionality. With the examination of the hysteretic loop in Figure 3, the accommodation property can be seen. The first and the fifth resistance curves are different. They are saturated at different resistance values. In order to examine the accommodation property, the resistance values of the heating curve were plotted at the same temperature point $T_{p}$ (ESD-ABS $T_{p}=$ 78 °C, ESD-PLA $T_{p}=$ 68 °C). We can see this accommodation in Figure 6 for several different loops. It can be recognized that the *R* values are decreased exponentially at the fixed *T* values as the number of excitation loops are increased. To derive the asymptotic line, the following curves were fitted to the decreasing resistance curves.

(1) $$R=m\cdot e^{b\cdot n}+a$$

where *R* is the resistance value in kΩ, *m* and *b* are parameters, *n* is the number of the loop, and *a* is a parameter as well. Since *b* is negative, the limit of the exponential curve is zero, so *a* will be asymptotic. Four different specimens were examined for the ESD-ABS and ESD-PLA as well. The mean value for the asymptotic of ESD-ABS composite is ${\bar{a}}_{ABS}=67.62$ kΩ with the deviation σ= 0.18 kΩ. For ESD-PLA, ${\bar{a}}_{PLA}=59.35$ kΩ with the deviation σ= 1.41 kΩ.

The addition of carbon to ABS or PLA composites can prevent or significantly decrease the occurrence of static electricity in the 3D printouts. While, for certain applications, this gained property of the ESD materials is useful, blending any basic raw composites with new substances can change the original properties of the samples. The dataset of the detailed DSC analysis shows well defined changes in many thermal parameters. The presence of carbon nanocomposites in several different host materials change the crystallization properties depending on the amount of the carbon filler [27,36,37,38,39,40]. The glass transition and melting phases remain relatively unchanged in most cases. Not just the presence of carbon particles but other additives and blending with other types of host materials may cause a dramatic change in certain properties of the composites [41,42]. It can also happen that the blending partner does not affect the crystallization parameters of the dominant host material significantly. Essentially, ABS does not really change the crystallization temperature characteristic for PBT (Poly(butylene terephthalate)) when blended [43]. The presence of carbon nanotubes or fibers nucleate crystallinity and increase the rate of crystallization [37,38,39] examined at 5 °C/min, 10 °C/min, 50 °C/min, and 100 °C/min heating rates (under nitrogen) while the mechanical properties of the fiber may remain unaffected [27]. The size of the fillers does matter as the smaller diameter nanotubes promote crystallization to a greater extent than the larger ones [44]. The presence of carbon can result in a sharper and narrower crystallization peak (means reduced time for crystallization completion) with higher amplitude that suggests a narrower crystallite size distribution in the composite [27] together with improved nucleation and crystal growth in the PLA matrix [36], which correlates well with our data, especially for the PLA samples (Figure 5). A narrower melting peak is also observable in the case of some nanocarbon-fiber composites at 10 °C/min to 50 °C/min heating rates [27,45] in which the effect is definitely present in the case of our ABS samples (Figure 4), but not clear for the PLA samples. Literature data confirms that the addition of carbon nanotubes can increase the crystallisation temperature for certain host materials, like PP (polypropylene) or PA6 (polyamide 6) [39,46], and decrease the melting enthalpy [41]. We could observe the same behaviour in the case of the ESD-ABS sample (both crystallization temperature increase, and melting-phase enthalpy decrease) while the PLA interestingly changed in the opposite direction. Although it is important that single-walled carbon nanotubes often aggregate into bundles and, in such a case, they lose the ability to nucleate crystal formation [44].

It seems that the addition of 10–20% carbon filler to either PLA or ABS host materials induce changes in the thermal parameters (especially in crystallization, melting, and decomposition phases) of the composites. However, an alteration that would significantly affect the thermal behaviour of these compounds during and after 3D printing was not observed (Figures S1–S4 and S6). The applied filler had a significant effect on the mechanical properties and the electrical conductivity of the PLA and ABS filaments [33]. Working with these ESD compounds is beneficial for the developers as the whole printing process of a biomedical sensor can be performed under the same circumstances and instrument settings. This quality makes the printed medical devices more reliable with both insulator and conductive components having very similar thermal and mechanical properties. The application of these ESD materials can be cost-effective in almost all applications in 3D printing because of the previously described properties.

**Figure 7:**
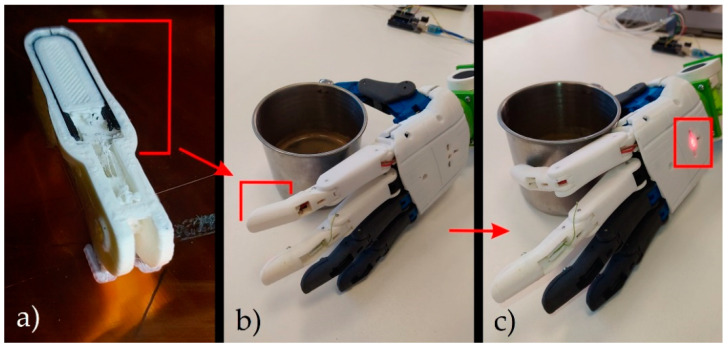
Image (**a**) shows the ESD-ABS printed in a black color. Image (**b**) shows the prosthetic fingertip with 3D printed ESD-ABS as a temperature sensor at an ambient temperature. Image (**c**) indicates the conductive fingertip touching the 50 °C cup and the alert LED lights up, indicating that it is a high temperature object.

Increasing the temperature, the ESD-ABS composite printed in the prosthesis showed a greater change in resistance than the ESD-PLA, so it has greater sensitivity, and, therefore, the current temperature values can be determined more reliably. Since the prosthesis is made of PLA, it can easily deform above 60 °C. In everyday life, the user may touch hot objects (like hot coffee) with the prosthesis several times, even without knowing the hot temperature of the object, which can cause permanent damage to the printed parts. Figure 7 image (a) shows the ESD-ABS printed in a black color, and image (b) shows the prosthetic fingertip with 3D-printed ESD-ABS as a temperature sensor in ambient temperature. Image (c) indicates the conductive fingertip touching the 50 °C metal cup and the alert LED lights up, indicating that it is a high temperature coffee cup.

## 5. Conclusions

Electrically conductive ABS-based and PLA-based carbon composites are suitable for electrical prototyping. Using dual-extruder 3D printers, complex models and objects can be manufactured, containing electrically conductive components. It is proven that the addition of 10–20% (*m*/*m*) carbon to ABS and PLA polymers does not have an effect on thermal characteristics. Therefore, it can be printed without limitations on thermal settings. Based on the results of the study, the electrical characteristics can be defined in the design phase of the model before the printing process. The printouts are suitable for sensor production and resistance measurements. Thus, these electronic components (sensors) can be essential in medical-robotic device developments, according to the electrical characteristics of the printouts. Potential areas of use are interventional and diagnostic robotic solutions, prosthesis and active orthosis developments, and health simulator developments. Applying the ESD-ABS composite printed thermometer sensor in a prosthesis is more reliable than the ESD-PLA because it has a greater temperature-resistance sensitivity. Therefore, in the case of a unit temperature change, it has a larger resistance change, and it allows a more accurate temperature measurement.

## Figures and Tables

**Table 1:** Corresponding temperature values of the characteristic DSC peaks in the heating cycle of different PLA and ABS samples (where *T_on_* is the initial temperature, *T_g_* is the glass transition temperature, and *T_end_* is the final temperature of the phase; *T_c_*, *T_m_* and *T_d_* stand for crystallization, melting and decomposition temperatures, respectively; *T_endo_* shows an extra endotherm peak). The errors are standard errors of means from at least three independent measurements. It is known that, because of the amorphous nature of ABS, it has no specific melting point, and finding the exact glass transition temperature is also difficult [34,35]. These values in the table are estimations based on the common pattern of both ABS composites in the given region. Please find the data of this table as a plot in the Supplementary Materials (Figure S6).

	Heating (30–600 °C)						
	Peak 1			Peak 2	Peak 3	Peak 4	Peak 5
	Glass Transition			Crystallization	Melting	Decomposition	Extra Peak
	*T_on_* (°C)	*T_g_* (°C)	*T_end_* (°C)	*T_c_* (°C)	*T_m_* (°C)	*T_d_* (°C)	*T_endo_* (°C)
PLA	61.85 ± 0.85	65.62 ± 0.17	68.51 ± 0.19	126.05 ± 0.88	151.60 ± 0.31	369.27 ± 0.62	-
ESD-PLA	49.80 ± 0.20	60.46 ± 0.04	64.32 ± 0.07	104.61 ± 0.60	153.90 ± 0.11	350.34 ± 0.44	402.76 ± 0.35
ABS	97.36 ± 0.09	103.89 ± 0.04	108.84 ± 0.22	119.40 ± 0.98	130.70 ± 0.42	423.03 ± 0.45	-
ESD-ABS	97.49 ± 0.09	108.11 ± 0.42	121.28 ± 0.42	130.03 ± 0.23	133.76 ± 0.28	420.14 ± 0.03	-

**Table 2:** Enthalpy change values of the characteristic DSC peaks in the heating cycle of different PLA and ABS samples. The errors are standard errors of means from at least three independent measurements. Please find the data of this table as a plot in the Supplementary Materials (Figure S6).

	Δ*H* Peak Enthalpies (J/g)—Heating (30–600 °C)				
	Peak 1	Peak 2	Peak 3	Peak 4	Peak 5
	Endotherm	Exotherm	Endotherm	Endotherm	Endotherm
PLA	12.72 ± 0.59	−3.25 ± 0.12	14.55 ± 0.71	1016.18 ± 26.49	-
ESD-PLA	18.58 ± 2.55	−18.49 ± 1.36	22.60 ± 0.96	425.85 ± 18.46	42.36 ± 0.76
ABS	2.55 ± 0.50	−6.22 ± 0.93	5.48 ± 0.69	647.15 ± 16.79	-
ESD-ABS	6.57 ± 0.42	−4.19 ± 0.11	2.58 ± 0.09	431.70 ± 5.14	-

**Table 3:** Corresponding temperature and enthalpy change values of the characteristic DSC peaks in the cooling cycle of different PLA and ABS samples (where *T_on_* is the initial temperature, *T_m_* is the recrystallization phase peak temperature, and *T_end_* is the final temperature of the phase). The errors are standard errors of means from at least three independent measurements.

Cooling (300–30 °C)					
Peak 6 (Recrystallization)					
Exotherm					
	*T_on_* (°C)	*T_m_* (°C)	*T_end_* (°C)	Extra *T_m2_* Peak (°C)	Δ*H* Peak Enthalpies (J/g)
PLA	117.34 ± 1.95	63.75 ± 0.20	49.95 ± 1.41	-	−10.16 ± 2.75
ESD-PLA	53.04 ± 0.16	49.22 ± 0.07	38.48 ± 1.23	-	−7.97 ± 0.20
ABS	135.18 ± 1.85	124.58 ± 0.78	92.04 ± 3.10	-	−5.79 ± 0.37
ESD-ABS	132.31 ± 1.04	123.77 ± 0.11	89.32 ± 5.47	107.63 ± 0.14	−6.06 ± 1.38

## Supplementary Materials

The following are available online at https://www.mdpi.com/2073-4360/12/12/2960/s1. Figure S1: Mass change of the untreated ABS sample during heating (from 30 °C to 300 °C) and cooling (from 300 °C to 30 °C), Figure S2: Mass change of the ABS-ESD sample during heating (from 30 °C to 300 °C) and cooling (from 300 °C to 30 °C), Figure S3: Mass change of the untreated transparent PLA sample during heating (from 30 °C to 300 °C) and cooling (from 300 °C to 30 °C), Figure S4: Mass change of PLA-ESD sample during heating (from 30 °C to 300 °C) and cooling (from 300 °C to 30 °C).
