# Supplementary material for: Detailed Thermal Characterization of Acrylonitrile Butadiene Styrene and Polylactic Acid Based Carbon Composites Used in Additive Manufacturing
Source: Polymers (Basel). 2020 Dec 11;12(12):2960. doi: 10.3390/polym12122960 (PMC7764123; doi:10.3390/polym12122960)
Supplement: Supplementary file 1 [file polymers-12-02960-s001.pdf]

Supplementary Materials of

Detailed Thermal Characterization of ABS and PLA Based Carbon Composites Used in Additive Manufacturing

Zoltan Ujfalusi [1], Attila Pentek [2], Roland Told [2], Adam Schiffer [3], Miklos Nyitrai [1,4] and Peter Maroti [1,2,5,*]

[1] Department of Biophysics, Medical School, University of Pécs, Szigeti str. 12, H-7624 Pécs, Hungary; zoltan.ujfalusi@aok.pte.hu (Z.U.); miklos.nyitrai@aok.pte.hu (M.N.)
[2] 3D Printing and Visualization Centre, University of Pécs, Boszorkany str. 2, H-7624 Pécs, Hungary; pentekattilaistvan@gmail.com (A.P.); told.roland@pte.hu (R.T.)
[3] Department of Technical Informatics, Faculty of Engineering and Information Technology, University of Pécs, Boszorkany str. 2, H-7624 Pécs, Hungary; schiffer.adam@mik.pte.hu
[4] MTA-PTE Nuclear-Mitochondrial Interactions Research Group, H-7624 Pécs, Hungary
[5] Medical Simulation Education Center, Medical School, University of Pécs, Szigeti str. 12, H-7624 Pécs, Hungary
* Correspondence: peter.maroti@aok.pte.hu; Tel.: +36-304426841

Received: 16 November 2020; Accepted: 9 December 2020; Published: date

Measurements were performed to see whether the examined samples suffer any decrease in their mass during 3D printing. Mass change for all four sample types was recorded at lower maximum temperature measurements (Figures S1-S4). The most important temperature values are marked on each curve. With the exemption of the ESD-PLA sample, all other composites lost a maximum 2% (m/m) from their initial mass when they were heated up to 300°C (furnace temperature). If we focus on the typical printing temperatures of these composites (~215 °C for the PLA and ~250 °C for the ABS samples), then there is no significant mass decrease for the samples.

Supplementary Figures

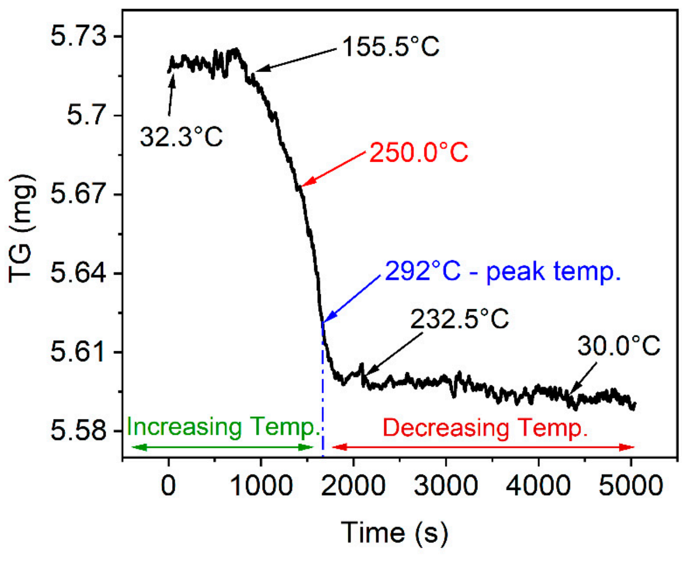

Figure S1. Mass change of the untreated ABS sample during heating (from 30 °C to 300 °C) and cooling (from 300 °C to 30 °C).

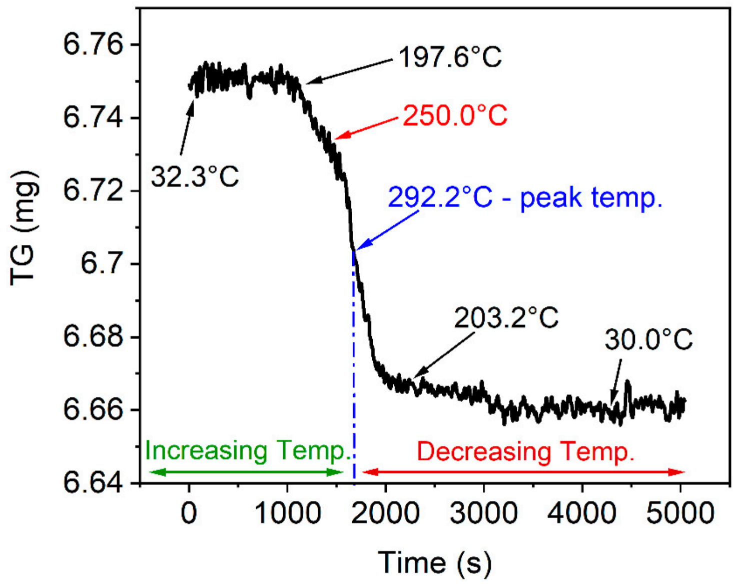

Figure S2. Mass change of the ABS-ESD sample during heating (from 30 °C to 300 °C) and cooling (from 300 °C to 30 °C).

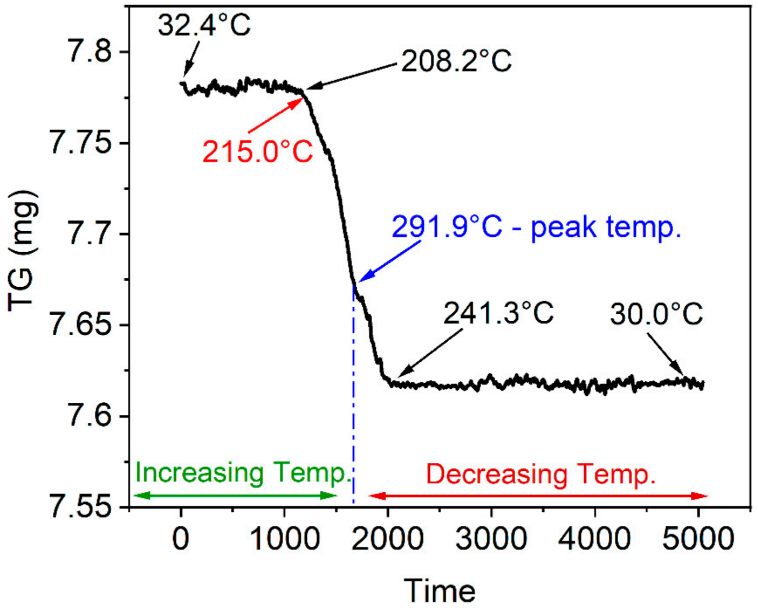

Figure S3. Mass change of the untreated transparent PLA sample during heating (from 30 °C to 300 °C) and cooling (from 300 °C to 30 °C).

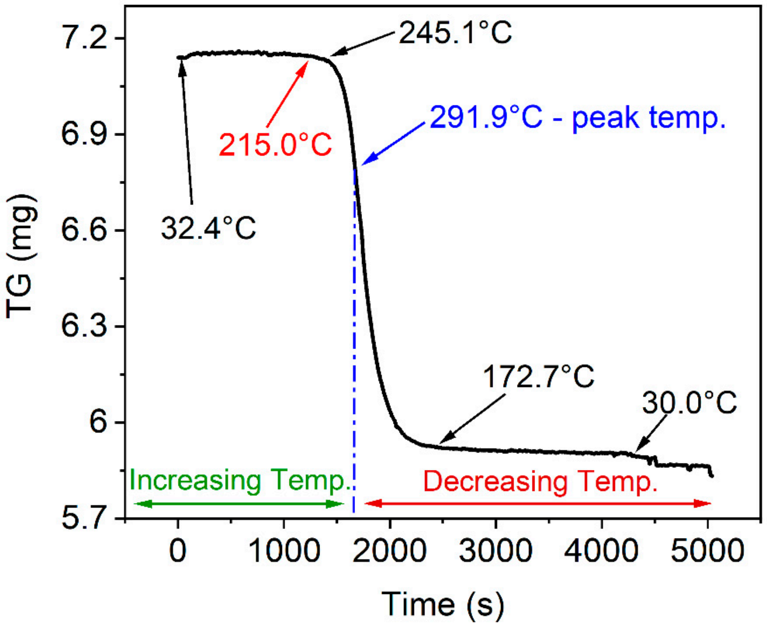

Figure S4. Mass change of PLA-ESD sample during heating (from 30 °C to 300 °C) and cooling (from 300 °C to 30 °C).

Publisher's Note: MDPI stays neutral with regard to jurisdictional claims in published maps and institutional affiliations.

© 2020 by the authors. Submitted for possible open access publication under the terms and conditions of the Creative Commons Attribution (CC BY) license (http://creativecommons.org/licenses/by/4.0/).
